# High Content Screening of a Kinase-Focused Library Reveals Compounds Broadly-Active against Dengue Viruses

**DOI:** 10.1371/journal.pntd.0002073

**Published:** 2013-02-21

**Authors:** Deu John M. Cruz, Andrea Cristine Koishi, Juliana Bosso Taniguchi, Xiaolan Li, Rafaela Milan Bonotto, Joo Hwan No, Keum Hyun Kim, Sungmin Baek, Hee Young Kim, Marc Peter Windisch, Ana Luiza Pamplona Mosimann, Luana de Borba, Michel Liuzzi, Michael Adsetts Edberg Hansen, Claudia Nunes Duarte dos Santos, Lucio Holanda Freitas-Junior

**Affiliations:** 1 Center for Neglected Diseases Drug Discovery (CND3), Institut Pasteur Korea, Seongnam-si, Gyeonggi-do, South Korea; 2 Instituto Carlos Chagas, Fundação Oswaldo Cruz Paraná (ICC/FIOCRUZ-PR), Curitiba, Paraná, Brazil; 3 Universidade Federal do Paraná (UFPR), Curitiba, Paraná, Brazil; 4 Universidade Estadual Paulista “Júlio de Mesquita Filho”, Araraquara, São Paulo, Brazil; 5 Image Mining Group (IMG), Institut Pasteur Korea, Seongnam-si, Gyeonggi-do, South Korea; 6 Universidade Feevale, Novo Hamburgo, Rio Grande do Sul, Brazil; 7 Applied Molecular Virology (AMV), Institut Pasteur Korea, Seongnam-si, Gyeonggi-do, South Korea; 8 Early Discovery Program, Institut Pasteur Korea, Seongnam-si, Gyeonggi-do, South Korea; Florida Gulf Coast University, United States of America

## Abstract

Dengue virus is a mosquito-borne flavivirus that has a large impact in global health. It is considered as one of the medically important arboviruses, and developing a preventive or therapeutic solution remains a top priority in the medical and scientific community. Drug discovery programs for potential dengue antivirals have increased dramatically over the last decade, largely in part to the introduction of high-throughput assays. In this study, we have developed an image-based dengue high-throughput/high-content assay (HT/HCA) using an innovative computer vision approach to screen a kinase-focused library for anti-dengue compounds. Using this dengue HT/HCA, we identified a group of compounds with a 4-(1-aminoethyl)-*N*-methylthiazol-2-amine as a common core structure that inhibits dengue viral infection in a human liver-derived cell line (Huh-7.5 cells). Compounds CND1201, CND1203 and CND1243 exhibited strong antiviral activities against all four dengue serotypes. Plaque reduction and time-of-addition assays suggests that these compounds interfere with the late stage of viral infection cycle. These findings demonstrate that our image-based dengue HT/HCA is a reliable tool that can be used to screen various chemical libraries for potential dengue antiviral candidates.

## Author Summary

Dengue, a re-emergent human disease that places nearly half of the world's population at risk, threatens to further expand in geographical distribution. The lack of an available effective dengue vaccine has encouraged the search for antiviral drugs as an alternative approach. In recent years, drug discovery through high-throughput screening has become a trend in the search for dengue antivirals. In this study, we developed an image-based dengue high-throughput/high-content assay using prevalent viral strains of three dengue serotypes (DENV1, DENV2 and DENV3) isolated from dengue outbreaks in South America and a laboratory-adapted strain of DENV4. We demonstrated the usefulness of our image-based dengue HT/HCA in identifying potential dengue antivirals by screening a small subset of chemical compounds for inhibition of dengue virus infection in a human-derived host cell line (Huh-7.5), and partially characterized their activities against dengue infection in a mosquito host cell line (C6/36), a distantly-related virus (hepatitis C virus), and an unrelated virus that is transmitted by the same mosquito vector (chikungunya virus).

## Introduction

Dengue virus (DENV) is an important mosquito-borne pathogen responsible for causing dengue fever (DF) and the more severe, life-threatening dengue hemorrhagic fever/shock syndrome (DHF/DSS) [1]. DENV is a small, enveloped virus belonging to the genus *Flavivirus*, family *Flaviviridae*. [2]. Dengue virions are approximately 50 nm in diameter [3], containing a single-stranded positive RNA of ∼11 kilobase with a genomic organization: 5′-C-preM-E-NS1-NS2A/B-NS3-NS4A/B-NS5-3′, and flanked by the 5′ and 3′ UTRs (untranslated regions) [4], [5]. There are 4 serotypes of dengue (DENV1, DENV2, DENV3, DENV4), with two or more serotypes commonly found to co-circulate in many dengue endemic areas [6], [7]. The presence of more than 1 dengue serotype in a geographical area contribute to the persistence of epidemics, as immunity acquired against one dengue serotype does not confer long-term protective immunity against heterologous serotypes [8]. Conversely, individuals that have acquired humoral immunity against one dengue serotype may be pre-disposed to DHF/DSS when subsequently infected with a heterologous serotype through antibody-dependent enhancement [9], [10]. Since its re-emergence in 1953, DENV has spread rapidly across 5 continents and more than 100 countries, mostly in tropical and subtropical regions. Present estimates by the World Health Organization place nearly 2.5 billion people at risk of dengue, with approximately 50 million cases of dengue infection and 20 thousand mortalities occurring annually [11].

Due to the global burden of dengue, numerous studies have been done to elucidate the nature of dengue infection and the underlying mechanisms of DF and DHF/DSS. Although ADE is the most widely accepted theory for the occurrence of DHF/DSS, several studies have suggested higher viremia titers, virus serotype and host genetic background as determinants of DHF/DSS [12], [13]. Extensive genetic, cellular and immunological studies have investigated the role of host innate immunity in the events leading to DHF/DSS [14]–[16]. However, a clear understanding on the immunopathology of dengue infections remains elusive.

With the number of DHF/DSS cases rising every year, the demand for a preventive, prophylactic, or therapeutic measure against DENV is growing rapidly. Significant progress has been achieved in the development of a dengue vaccine, including one vaccine candidate (ChimeriVax) that has passed through Phase II clinical trials [17]. However, its long-term efficacy and safety has not been established and mass production of this vaccine candidate to meet the growing demand remains a daunting task. A new approach that is gradually gaining interest is the development of dengue antivirals. For the last 6 years, several drug candidates for HCV and other RNA viruses have been pursued for repositioning as potential drug candidates for dengue [18]. Though at present, none of these compounds have gone beyond pre-clinical trials.

Recent advancements in high-throughput screening (HTS) technologies have contributed to increasing efficiency in the drug discovery process. These include *in silico* HTS, *in vitro* enzymatic assays, cell-based reporter assays, image-based whole infection assays, among others [19]. In the work reported here, we describe the development of an image-based high-throughput/high-content assay (HT/HCA) screening method for anti-dengue compounds using an infectious virus system and an innovative approach in image analysis. Using this dengue HT/HCA system, we screened a BioFocus kinase inhibitor library of 4,000 small molecules against DENV1-4 to identify compounds that possess antiviral activity against all 4 serotypes during infection of a human host cell. We counter-screened the primary hits against DENV infection in *Aedes albopictus* clone C6/36, hepatitis C virus (Family *Flaviviridae*, genus *Hepacivirus*) infection, and chikungunya virus (Family *Togaviridae*, genus *Alphavirus*) infection to partially characterize the compounds as having a host-specific or virus-specific target. The dengue hit compounds were clustered based on their chemical structures and, together with the activity profile, used to identify scaffolds whose antiviral activity against the 4 serotypes vary depending on the chemical substituents. These scaffolds identified from our dengue HT/HCA screening could be used as potential starting points for the development of dengue antivirals.

## Methods

### Cells and Viruses and Antibodies

The mosquito cell line C6/36 *Aedes albopictus* clone (CRL-1660), mouse hybridoma cells D1-4G2-4-15 (HB-112), and human hepatocyte Huh-7.5 (PTA-8561, U.S. Patent Number 7455969) were obtained from the American Type Culture Collection. HuH-7 (JCRB0403) was kindly provided by Dr. Katja Fink. Three South American isolates of dengue viruses: Den1 BR/90 (GenBank AF226685.2), BR DEN2 01-01 (GenBank JX073928), BR DEN3 290-02 (GenBank EF629369.1), and the World Health Organization laboratory strain DEN4 TVP-360 were generously provided by Dr. Claudia N. Duarte dos Santos. Hepatitis C virus (HCV) genotype 2a (JFH-1) expressing the NS5a-GFP fusion protein was kindly provided by Dr. Marc Windisch and the chikungunya virus (CHIKV-118-GFP) was a generous gift from Dr. Olivier Schwartz. C6/36 was maintained at 28°C in Leibovitz's L-15 media (Gibco/Invitrogen, USA) supplemented with 5% Fetal Bovine Serum (FBS, Gibco/Invitrogen, USA), 0.26% Tryptose Phosphate Broth (TPB, Sigma-Aldrich, USA) and 25 µg/mL Gentamicin Sulfate (Gibco/Invitrogen, USA) and passaged every 3–4 days. Huh-7.5 was maintained under humidified conditions at 37°C, 5% CO_2_ in Dulbecco's minimum essential medium/Hank's F-12 (DMEM/F12, 1∶1) (Gibco/Invitrogen, USA) supplemented with 10% FBS and 100 U/mL Penicillin/100 µg/mL Streptomycin (antibiotic solution, Gibco/Invitrogen, USA) and passaged every 3–4 days. HuH-7 was cultured under humidified conditions at 37°C, 5% CO_2_ in RPMI 1640 containing 25 mM HEPES (WelGene, South Korea) supplemented with 10% FBS and antibiotic solution. Low passaged dengue viruses were propagated for 7 days in C6/36 maintained at 28°C in Virus Medium (VM: Leibovitz's L-15 medium supplemented with 1% FBS, 0.26% TPB, 25 µg/mL Gentamicin) according to previously described methods [20] and titrated by focus formation assay (FFA) using C6/36 as previously described [21]. Dengue virus titers were expressed as focus forming units per mL (ffu/mL). Flavivirus group-specific αE monoclonal antibody 4G2 [22], used as detecting antibody, was prepared from culture supernatant of D1-4G2-4-15 maintained under humidified conditions at 37°C, 5% CO_2_ in RPMI 1640 containing 25 mM HEPES (WelGENE, South Korea) and supplemented with 10% FBS, 1 mM sodium pyruvate (Sigma-Aldrich, USA), antibiotic solution and 250 ng/mL Amphotericin B (Sigma-Aldrich, USA). 4G2 was concentrated by ammonium sulfate precipitation following previously described methods [23] and purified by protein G affinity chromatography (GE Amersham, Sweden) according to manufacturer's instruction. Total antibody protein was determined by spectrophotometric analysis using the formula of Warburg and Christian [24].

### Compound Library and Reference Compounds

A small target-focused chemical library, comprising of 4,000 synthesized compounds based on ligand binding of known kinase binding sites, was sourced from BioFocus (Galapagos, Belgium). Reference compounds were purchased from TOCRIS Bioscience (Bristol, UK): AZ 10417808, BIBU 1361, LE 135 and MPP and Sigma-Aldrich (USA): Chloroquine, ribavirin and recombinant human Interferon-αA (IFN-α2A). All compounds from the BioFocus kinase inhibitor library and reference compounds were prepared in 100% dimethyl sulfoxide (DMSO, Sigma-Aldrich, USA), with the exception of IFNαA that was prepared in Dulbecco's Phosphate-buffered saline (DPBS, WelGENE, South Korea) containing 5% FBS.

### Liquid Handling and Automation

For dispensing of liquid media containing the host cell and viruses, the Thermo Scientific WellMate (Fischer Brand, USA) was used. Dispensing of antibody solutions and other liquid reagents for IFA, including the washing steps, was done using the 96/384-head BioTek EL406 automated liquid washer/dispenser (BioTek, USA).

### Assay Miniaturization and Optimization

For miniaturization of the image-based dengue HT/HCA, the following conditions were optimized: a) host cell seeding density, b) multiplicity of infection (M.O.I.) and c) incubation period of infection. For the optimum host cell density, Huh-7.5 cells were prepared at various cell densities and seeded in a 384-well plate, μ-clear black (Greiner Bio-one, Germany). The cells were cultured between 2–4 days at 37°C, 5% CO_2_. For dengue virus infection, the optimum cell seeding density of Huh-7.5 was inoculated with DENV1, DENV2, DENV3 or DENV4 at various M.O.I. (0.1–5) and cultured between 2–4 days at 37°C, 5% CO_2_.

### Immunofluorescence Detection of Dengue-infected Cells

An immunofluorescence assay (IFA) used to detect dengue infection was optimized for the dengue HT/HCA. Briefly, cells were fixed with 4% (w/v) paraformaldehyde (PFA) for 20 min at room temperature (Rm T). PFA-fixed cells were treated with 0.25% (v/v) Triton-X for 20 min at Rm T. DENV-infected cells were detected by probing with 4G2 mAb prepared in blocking buffer: DPBS containing 5% FBS for 30 min at 37°C, followed by AlexaFluor-conjugated goat anti-mouse IgG (H+L) (Invitrogen Molecular Probes, USA) prepared in blocking buffer for 30 min at 37°C. Cell nuclei counterstained with 5 µg/mL 4′,6-diamidino-2-phenylindole (DAPI, Sigma-Aldrich, USA). Two washing cycles of DPBS was done after each step of the IFA. After the final washing, digital images were acquired using a high-throughput confocal fluorescence imaging system (Evotec Technologies High-Throughput Cell Analyzer Opera, Perkin Elmer, USA). The digital images were taken from 3 different fields of each well at 20× magnification.

### Image Analysis Software Development

Acquired images were analyzed using our in-house developed image-mining platform (IM). This platform is designed to do high-content screening and directly access the database of images that were sequentially analyzed with specially designed algorithms developed as a customized plug-in to the IM platform. The results of all the analyses were stored in a centralized database. The IM plug-in for dengue HT/HCA works by independently analyzing two separate channels acquired with the Evotec Technologies High-Throughput Cell Analyzer Opera using different algorithms and converging these results to yield the final readout. One channel (DAPI-channel) captures the signal emitted by DAPI-stained nuclei at 450 nm, while the other channel (A488-channel) captures the signal emitted by the AlexaFluor 488 dye bound to the dengue E protein-antibody complexes confined in the cytoplasm of dengue-infected cells at 540 nm. To define the percentage of dengue-infected cells and, conversely the non-infected cells, a modified watershed method was applied. Compared to the original watershed algorithm [25] that uses the morphological gradient image, a weighted gradient image is used for the topographic surface, whose weights are defined as the ridge values computed from the eigen values of the original image. The percentage of non-infected cells, which is also defined as the percent inhibition or percent activity, is derived by using the formula: [1−(A488-positive cells/total cells)]×100%.

### Assay Validation with Reference Compounds

The dengue HT/HCA was validated by 1) by infecting Huh-7.5 cells in 384-well plates spotted with 0.5% DMSO with DENV or MOCK plated and 2) observing the dose-response curves of reference compounds previously reported to have anti-dengue activity. In both validation experiments, Huh-7.5 was mixed with DENV1, DENV2, DENV3 or DENV4 at a M.O.I. of 0.5 or VM only for MOCK-infection and dispensed in designated wells using the Thermo Scientific WellMate automated liquid dispenser. For the first validation experiment, the statistical reliability of the dengue HT/HCA was determined by calculating the Z'-factor for the percent infection or percent inhibition. Briefly, the Z'-factor of a defined parameter is calculated using the formula: 1−[(3*σp*+3*σn*)/(|*μp*−*μn*|)], where the *μp*, *μn*, *σp* and *σn* are the means (*μ*) and standard deviations (*σ*) of the positive (*p*) and negative (*n*) controls [26]. For the second validation experiment, the reference compounds were prepared in 2-fold serial dilutions and dispensed in duplicate wells in the 384-well plate, followed by the Huh-7.5 and DENV mixture (M.O.I. 0.5). Percent infection, percent inhibition, and percent cell number were determined using the customized IM platform plug-in. Scatter-plot distribution was generated using TIBCO Spotfire 4.5.0 (TIBCO Software Inc., Somerville, MA). The ten-point dose-response curves (10-pt DRCs) were plotted using the non-linear regression formula: log (inhibitor) vs. response – variable slope (4 parameters), available in GraphPad Prism 5.04 (GraphPad Software Inc., San Diego, CA). The top and bottom values of the reference compounds were unconstrained when the curve fittings of the 10-pt. DRCs were generated.

### Primary Screening of the BioFocus Kinase Inhibitor Library

The BioFocus kinase inhibitor library were screened against DENV1, DEN2, DENV3 and DENV4 at 10 µM in 0.5% (v/v) DMSO. MOCK-infected Huh-7.5 and IFN-α2A (500 U/mL) were used as positive controls, and the 0.5% DMSO vehicle was used as negative control. Four sets of 15 384-well plates (13 plates designated for test compounds and 2 plates designated for DMSO vehicle control) were used for the primary screening of the BioFocus kinase inhibitor library against each dengue serotype. Each of the 4,000 compounds in the library was tested in single wells. For data normalization and quality control of the screening, each test compound plate contained 16 replicates of the positive and negative controls. After dispensing the test compounds IFN-α2A and DMSO vehicle in the 384-well plates, Huh-7.5 was mixed with DENV1, DENV2, DENV3, or DENV4 to achieve a M.O.I. of 0.5 and dispensed at 5×10^3^ cells/well using the Thermo Scientific WellMate automated liquid dispenser. For the MOCK-infected Huh-7.5, the cells were mixed with VM and dispensed under the same conditions as previously stated. Virus infection in the presence of the compounds proceeded at 37°C, 5% CO_2_ for 96 hrs. Compound activity based on percent inhibition and cell toxicity was assessed by IFA and IM analysis as described above. Scatter-plot distribution of the entire screening was generated using TIBCO Spotfire 4.5.0 (TIBCO Software Inc., Somerville, MA).

### Data Normalization and Assay Quality Control

The calculated activity was normalized to a percent inhibition (PI) based on the MOCK-infected cells (100% activity, or zero infection) and dengue-infected cells (0% activity, or maximum measured infection percentage) controls according to the formula:
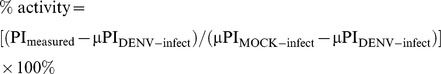
where: PI_measured_ - percent inhibition of test compound

μPI_DENV-infect_ - average percent inhibition readout of dengue-infected control

μPI_MOCK-infect_ - average percent inhibition readout of non-infected control

Similarly, the percent cell number was normalized based on the measured cell number in MOCK-infected (100% cell number) controls according to the formula: % cell number = (C_measured_/μC_MOCK-infect_)×100%, where C_measured_ is the measured cell number in the test well and μC_MOCK-infect_ is the average cell number in the MOCK-infected controls. The statistical validity of the dengue high-throughput screening was determined by calculating for the Z'-factor using the 0.5% DMSO-treatment and MOCK-infected Huh-7.5 as negative and positive controls, respectively. In addition, other parameters, including DRC of a reference compound, were used to evaluate the assay performance. For the primary screening a Z'-factor ≥0.5 and a coefficient of variation (CV) among the controls ≤10% was used to validate the results of the assay. The hit (i.e. a compound that demonstrates inhibition of infection) selection criteria for the primary screening was set at ≥80% inhibition of dengue viral infection in at least 1 dengue serotype and with the corresponding percent cell number at ≥50%.

### Counter-screening against Dengue Infection in C6/36

The hits identified from the primary screening were tested at 10 µM for inhibition of dengue virus infection in C6/36. Briefly, cells were inoculated with DENV1∼4 at an M.O.I. of 0.5 and seeded in 384-well plates spotted with the reference and primary hit compounds and incubated for 96 hrs at 28°C. Detection of dengue-infected cells by IFA and image acquisition using Evotec Technologies High-Throughput Cell Analyzer Opera was carried out following the method described above. Compound activity was determined by measuring percent inhibition and percent cell toxicity using the IM platform as previously described.

### Counter-screening against HCVcc

The hits identified from the primary screening were tested at 10 µM against HCV genotype 2a (JFH-1) infection in Huh-7.5 using an *in vitro* HCV cell culture system (HCVcc). Cells were seeded in 384-well plates and cultured under humidified conditions at 37°C for 24 hrs. Reference and primary hit compounds were added, followed by inoculation with HCV at an M.O.I. of 1 and incubated for another 72 hrs at 37°C. HCV-infected cells were identified by detection of NS5A-GFP expression using ImageXpress Ultra (Molecular Devices, USA) and analysis using the IM platform as previously described. Compounds resulting in ≥50% inhibition of HCV genotype 2a infection and percent cell number ≥50% were considered as positive hits for anti-HCV activity.

### Counter-screening against Chikungunya Virus

The hits identified from the primary screening were also tested at 10 µM against CHIKV-118-GFP infection in HuH-7 cells and evaluated by resazurin reduction assay (RRA). Resazurin (7-Hydroxy-3*H*-phenoxazin-3-one 10-oxide) is reduced to the red fluorescent resorufin by redox enzymes produced by viable cells, and is a good indication of metabolic capacity, and by extension cell viability. The amount of converted resorufin was measured as relative fluorescence readout (RFU) at excitation/emission of 531/572 nm using a fluorescence spectrophotometer (Victor^3^ V Spectrophotometer, Perkin Elmer, USA). Briefly, cells were inoculated with CHIKV-118-GFP at an M.O.I. of 0.5 and seeded in 384-well plates containing reference and primary hit compounds and incubated under humidified conditions for 72 hrs at 37°C. Resazurin solution was added to a final concentration of 10 µM and further incubated for another 12 hrs prior to measurement of RFU. The percent activity of the compounds, reflected by the percent cell viability, was quantified by normalizing against the RFUs of MOCK-infected cells and CHIKV-118-GFP-infected cells. Compounds resulting in normalized RFU ≥70% were considered as positive hits for anti-CHIKV activity.

### Hit Confirmation by Dose-response Curves

To confirm the compound activity against dengue viruses, the selected hits from the primary screening were tested in a 10-pt. DRC (2-fold serial dilution from 50 µM) using the same assay described for the dengue HT/HCA. Each concentration of the hit compounds was tested in duplicate wells. Data generated from image analysis of the 10-pt. DRC was plotted and analyzed using the non-linear regression formula: log (inhibitor) vs. response – variable response (4 parameters) in GraphPad Prism 5.04. The EC_50_ value, defined as the effective concentration resulting in a 50% inhibition of DENV infection, was used to evaluate compound activity. Compound toxicity was determined by testing the hit compounds in a 10-pt. DRC against Huh-7.5 in the absence of viral infection and measuring the cell viability using resazurin reduction assay as described above. The CC_50_ value, defined as the compound concentration resulting in a 50% reduction in cell viability (based on normalized RFU values) compared with the MOCK-infection, was used to evaluate cell toxicity. Confirmed hits were selected based on their *Selectivity Index* (SI), a dimensionless value that indicates the magnitude between cytotoxic concentration and effective concentration, and is calculated as: SI = CC_50_/EC_50_.

### Structural Analysis of Hit Compounds

Cluster analysis was done using a molecule-clustering module from Pipeline Pilot (Accelrys Software Inc., San Diego, CA, USA). The active scaffolds of compounds confirmed to have anti-dengue activity through dose response curves were selected for structural analysis. Structural relationship among the hit compounds was analyzed using the Tanimoto coefficient structural similarity [27].

## Results

### High Throughput-High Content Assay Development

Several phases were involved in developing the image-based dengue high-throughput/high-content assay (HT/HCA). A schematic workflow diagram of the assay development and assay method is shown in Figure S1. The first phase involved miniaturization of the assay to the 384-well plate format, including host cell seeding density and viral infection conditions. Selection criteria for the appropriate cell seeding density was included having a sufficiently high number cells but with enough spatial distribution for proper identification and accurate segmentation by the IM platform plug-in. After testing various seeding densities of Huh-7.5, the seeding density of 5×10^3^ cells per well was selected (data not shown). DENV infection in Huh-7.5 was visualized by immunofluorescence assay (IFA) detection of the dengue E protein using the 4G2 mAb and confocal imaging using the Evotec Technologies High-Throughput Cell Analyzer Opera. For the DENV infection, a M.O.I. of 0.5 and incubation time of 96 hrs was used since it allows for multiple rounds of virus replication and facilitates the screening of active compounds that target different stages of the dengue virus life cycle (Figure S2).

### Developing the IM Plug-in for Dengue HT/HCA

**Figure 1:**
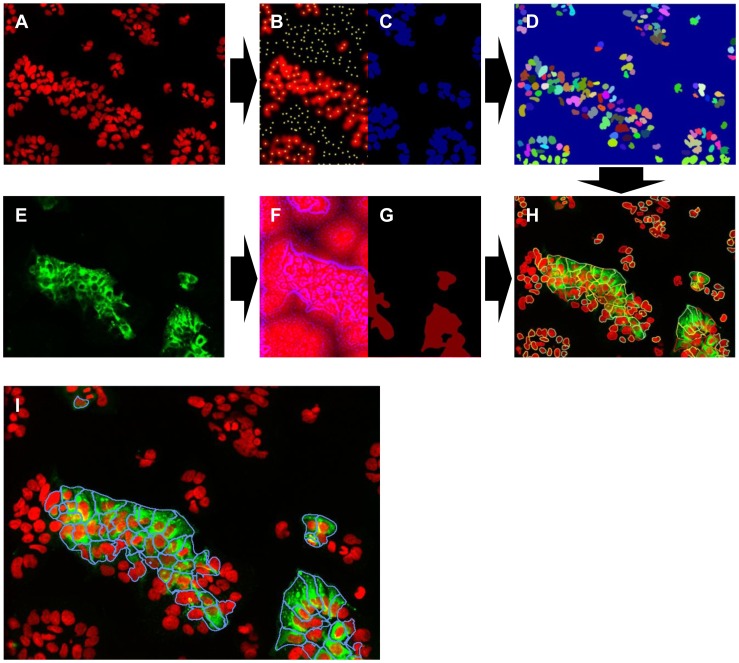
Software development for image-based analysis of dengue virus infection. Acquired confocal images of dengue infection in Huh-7.5 were analyzed using an in-house IM software. The number of cells in the images is determined by detecting the local maxima (B and C) from the DAPI channel (A). The region of a single nucleus (D) is defined using watershed-like method. Afterwards, a weighted distance map (F and G) of Alexa488 signal channel (E) is calculated to direct further watershed from the border of detected nucleus border (D) to define the so-called cell body. Finally, quantitation of dengue-infected cells is done by calculating the number of Alexa488-positive cell cytoplasm (the blue-marked cells in I) divided by the total number of cells (all the colorful-marked cells in H). (All images were taken at 20× magnification, false-color).

A flowchart of the image analyses is shown in Figure 1. Defining the cell nuclei was done as follows (Figure 1A–D): after applying a Gaussian low pass filter [28] with relatively high sigma value (to the nucleus size) on the DAPI channel (Figure 1A), the local maxima (Figure 1B) were subsequently located. A k-means clustering method [29] was then utilized to separate the background and foreground to obtain the nuclei mask image (Figure 1C) and the local maxima located in the background were removed, leaving the remaining maxima as those representing the number of cells in the image. Starting from the local maxima constrained by the nuclei mask, along with the slightly blurred nucleus image as a distance map, the region for single nuclei were defined (Figure 1D) with the watershed method [25].

Identifying the dengue-infected cells and the percentage of non-infected cells were done as follows (Figure 1D–I): from the image obtained from the A488-channel (Figure 1E), a weight map was calculated based on the edge features of this channel (Figure 1F). After applying an open-by-reconstruction operator and Gaussian low-pass filter to alleviate the noise, a foreground mask was attained (Figure 1G). Starting from the separated nuclei borders (Figure 1D) constrained by the foreground mask, and along with the weight map, the region of the signals were defined and marked with four different colors (Figure 1H), delineating the borders of the cells. Finally, the dengue-infected cells are identified as those having an A488 signal within the defined cell borders above a pre-defined threshold level, and are delineated by blue line segments (Figure 1I).

### Assay Validation

**Figure 2:**
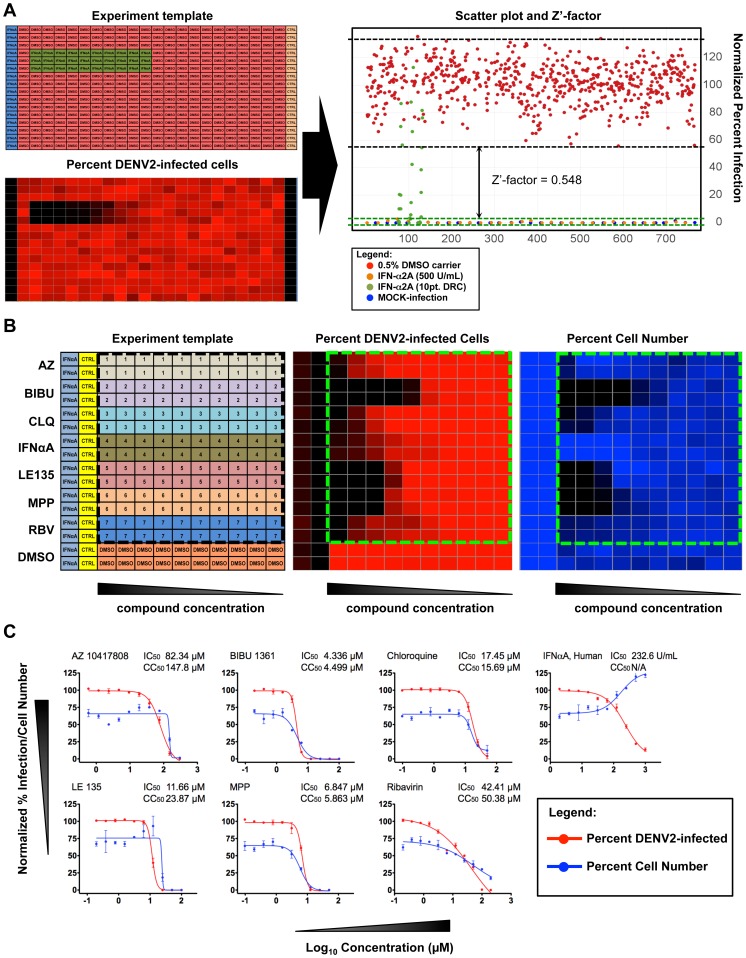
Assay validation of the image-based dengue HT/HCA. The first assay validation determined the degree of separation between positive and negative controls (A). *Upper left panel*: layout of controls in the 384-well plate; *Lower left panel*: generated heat-map representing percentage of Huh-7.5 infected with DENV2; *Right panel*: Scatter plot distribution shows the percentage of DENV2-infected cells under the following treatments: 0.5% DMSO carrier (red), 500 U/mL IFN-α2A (orange), 10 pt. DRC of IFN-α2A (green), MOCK-infection (blue). Area under the black and green dotted lines represents the variability of DENV infection and MOCK infection controls, respectively. The arrows represent the degree of separation (Z'-factor) between the two controls. The second assay validation determined the dose-response curves of a reference compound panel (B). *Left panel*: layout of the reference compounds spotted in designated wells of a 384-well plate; *Middle panel*: heat-map showing relative percentage of DENV2-infected cells; *Right panel*: heat-map showing relative percent cell viability based on average cell number. (C) 10 pt. DRC of the reference compound panel. Percent infection was normalized against DENV2-infected Huh-7.5, and percent cell number was normalized against MOCK-infected Huh-7.5.

The first assay validation evaluated the Z'-factors for DENV1-4 infection of Huh-7.5 in the 384-well plate format. Figure 2A shows a representation of the validation process done for the DENV2 HT/HCA. Cells, virus, and a reference control were dispensed in 384-well following a designed template pattern (upper left panel). After the viral infection period and IFA, IM analyses of the acquired images revealed the infection percentage, cell number based on nuclei detection and other pre-defined parameters. An IM analysis showing the relative percentage of DENV2 infection is represented by a generated heat map (lower left panel). The Z'-factor was calculated using the average and standard deviations of the percent infection of the positive and negative controls (right panel). MOCK-infected Huh-7.5 was designated as positive control while the DENV-infected Huh-7.5 was used as the infection control. All wells contained 0.5% DMSO vehicle to simulate the culture conditions used in the screening. The calculated Z'-factors for the DENV1, DENV2, DENV3, and DENV4 HT/HCA in 384-well plates showed a range between 0.50 and 0.75 (Figure S3). According to Zhang et al. [26] a Z'-factor ≥0.5 indicates a statistically reliable separation between positive and negative controls.

The second assay validation tested a panel of reference compounds previously reported to have antiviral properties against different strains of DENV2. This panel includes: AZ10417808, BIBU1316, MPP, LE 135 [30], Ribavirin [31], Chloroquine [32] and IFN-α2A [33]. MOCK-infection and 0.5% DMSO were used as positive and negative controls, respectively. The compounds' antiviral activities and cell toxicities against BR DEN2 01-01 infection of Huh-7.5 were determined by DRC. Figures 2B shows the generated heat map for percent DENV2-infected cells and percent cell viability. Figure 2C shows the DRC of the reference panel, with the percent infection normalized against DENV2-infected Huh-7.5 and percent cell viability normalized against MOCK-infected Huh-7.5. It was observed that all compounds in the reference panel showed inhibition of DENV2 infection in a dose-dependent manner. At very low concentration of the reference compounds, the percent cell viability of DENV2-infected cells did not exceed 75% compared with the MOCK-infected cells, as a consequence of DENV2-associated cytopathic effect. The resulting EC_50_ of the reference compounds against the DENV2 infection of Huh-7.5 using our dengue HT/HCA varied from those previously reported. Furthermore, most of the compounds in our reference panel exhibited significant cell toxicities at EC_50_ compared with the MOCK-infected and DENV2-infected controls. Conversely, IFN-α2A concentration ≥EC_50_ resulted in higher cell numbers compared with MOCK-infected Huh-7.5. Discrepancies between the EC_50_ of the reference compounds obtained in this study with the previous reports may be attributed to factors such as intrinsic differences between the DENV2 strains and the type of host cell used. Nonetheless, the results of the assay validation demonstrate the statistical reliability of our developed dengue HT/HCA. None of the compounds in the reference compound panel exhibited the ideal EC_50_ and CC_50_ values for use in the dengue HT/HCA. While IFN-α2A has shown strong antiviral properties against dengue infection, having a multi-target mode of action restricts its application as a reference drug. Based on these observations, MOCK-infection and IFN-α2A were used as positive controls for the screening of the compound library, but only MOCK-infection was used for calculating Z'-factors and validating the reliability of the entire screening process.

### Screening of the BioFocus Kinase Inhibitor Library

**Figure 3:**
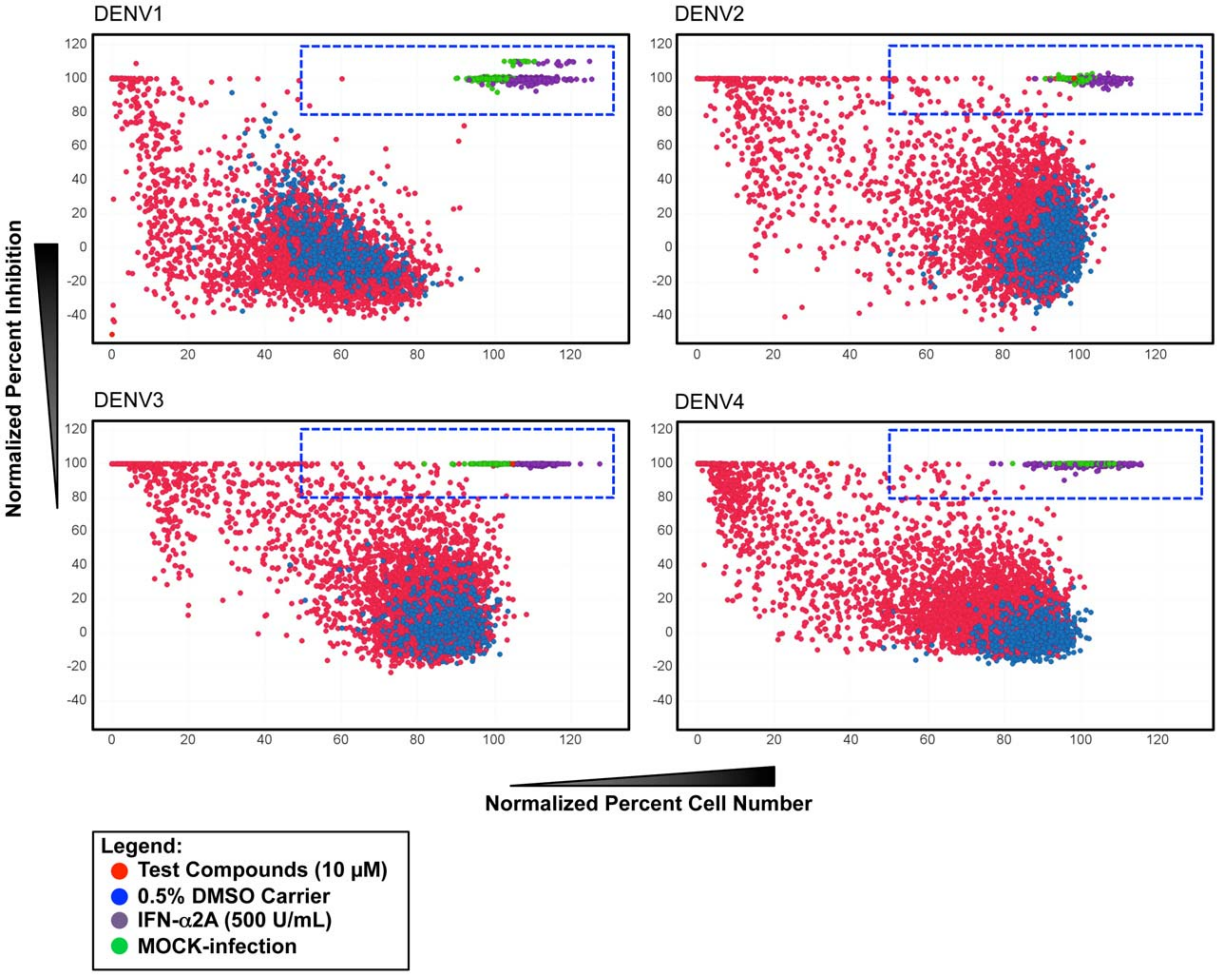
Primary screening of the BioFocus kinase inhibitor library. A collection of 4,000 compounds synthesized on the basis of known kinase binding sites were screened for antiviral activity against DENV1, DENV2, DENV3 and DENV4 infection in Huh-7.5. Scatter-plot distribution shows compound activity based on percent inhibition and percent cell number resulting from the following treatment: 10 µM test compounds (red), 0.5% DMSO carrier (blue), MOCK-infection (green), 500 U/mL IFN-α2A (purple). Area under the dotted squares indicate selected primary hits from each serotype based on the criteria: ≥80% inhbition and <50% cell number.

The compounds screened with our dengue HT/HCA is a subset of 4,000 small molecules belonging to the BioFocus kinase inhibitor library of chemical compounds designed to interact with one of the seven representative subsets of kinases according to protein conformations and ligand binding modes [34]. The library was screened at 10 µM against DENV1, DENV2, DENV3 and DENV4, and primary hits were selected based on the criteria: ≥80% activity and ≥50% cell number (Figure 3). The 50% cell number threshold was chosen to allow a wider range of compounds that are slightly cytotoxic at 10 µM, but may still be active at lower concentrations, to be selected. Primary hits were selected for activity against at least 1 dengue serotype. Out of the 4,000 small molecules screened, 157 compounds qualified for further confirmation and counter-screening, giving a hit rate of 3.9%. The primary hits were selected according to activity (≥80% inhibition), irrespective of their cytotoxicity levels. Among the 157 primary hits, 40 compounds (25.5%) showed inhibition of all 4 serotypes, 19 (12.1%) against 3 serotypes, 30 (19.1%) against 2 serotypes, and 68 (43.3%) against 1 serotype.

### Profiling the Activity of Dengue Primary Hits

The inhibitory properties of the dengue primary hits were further investigated by testing these compounds at 10 µM against DENV infection of C6/36, HCV genotype 2a infection of Huh-7.5, and CHIKV-118-GFP infection of HuH-7. The activity profile of these dengue primary hits is summarized in Figure S4. Thirty-nine of the dengue primary hits (24.8%) exhibited ≥50% inhibition against at least 1 DENV serotype in the C6/36 host, suggesting that the targets of these compounds are required for successful DENV infection in both human and insect host cells. It is important to note that even though the other 118 dengue primary hits (75.1%) did not inhibit DENV infection in C6/36 at the same concentration, the putative role of their targets in DENV infection in the insect cells have not been ruled out. Conversely, 103 dengue primary hits (65.6%) showed ≥50% inhibition of HCV genotype 2a infection of Huh-7.5 at 10 µM, with only 19 hits exhibiting <50% cell number in the host cell. In contrast to the high number of overlapping hits between DENV and HCV genotype 2a, only 9 (5.7%) of the dengue primary hits exhibited detectable activity against CHIKV-118-GFP in the resazurin reduction assay. These hits had low antiviral activity, and were excluded after conducting DRC analysis (data not shown).

### Chemical Structures of Anti-Dengue Compounds

**Figure 4:**
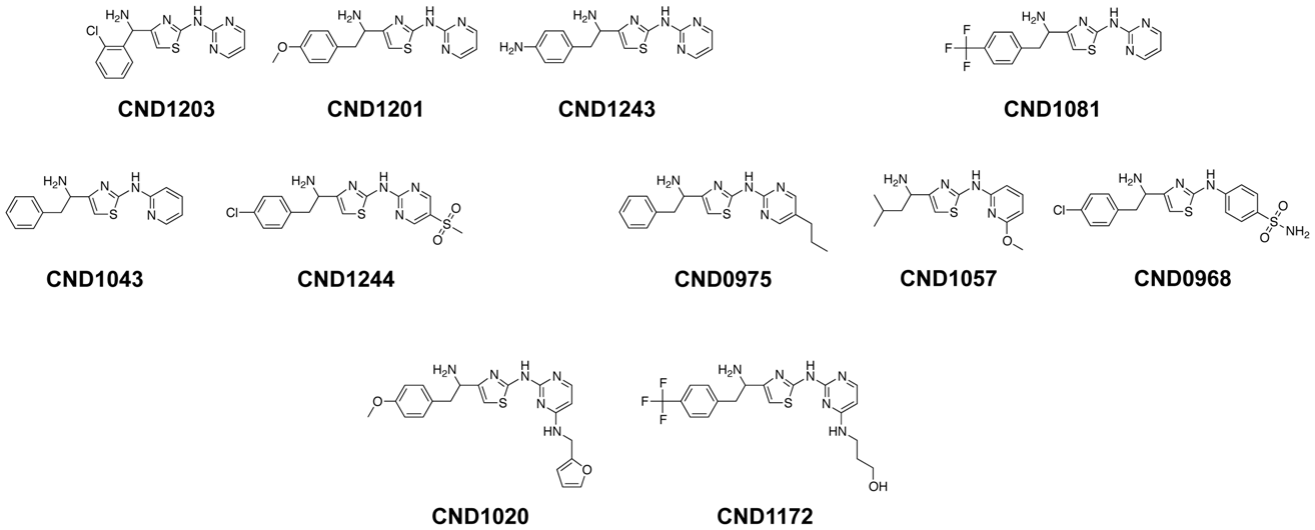
Chemical structures of the 11 compounds having the 4-(1-aminoethyl)-N-methylthiazol-2-amine core scaffold.

The activities of the 157 primary hits were confirmed by 10 pt. DRC against DENV1, DENV2, DENV3 and DENV4 infection in Huh-7.5. Cluster analysis of the top 53 compounds exhibiting the lowest EC_50_ values were performed using a molecule-clustering module from Pipeline Pilot yielded 4 enriched clusters plus singletons. Core structures of the two scaffolds were heterocyclic ring of imidazopyridine and the other two scaffolds were thiazole-based compounds. One of the thiazole scaffold clusters, consisting of 11 compounds, had 4-(1-aminoethyl)-N-methylthiazol-2-amine as a common core structure. The profile of these compounds (EC_50_, CC_50_ and Selectivity Index) against the four dengue serotypes and their chemical structures are shown in Table 1 and Figure 4, respectively. The compounds showing a wide spectrum of anti-dengue activity against all four serotypes have only pyridine or pyrimidine ring by amine linkage to the core scaffold and addition of substituents on the ring narrows the spectrum of activity, especially against DENV4. In addition, 9 out of 10 compounds in this cluster have an extra carbon next to aminoethyl linkage at 4th position of thiazole, followed by a phenyl group and trifluoro-, methoxy-, amine or chloride substituents on *para* position of the phenyl group.

**Table 1:** Serotype specificity and virus selective of the hit compounds in the 4-(1-aminoethyl)-N-methylthiazol-2-amine cluster.

Specificity					EC_50_ (µM)/SI				
(Serotype)				Compound	CC_50_ (µM)	DENV1	DENV2	DENV3	DENV4
1	2	3	4	CND1203	>100	1.7/>59	4.1/>24	2.3/>43	5.0/>20
1	2	3	4	CND1201	>100	0.9/>111	7.6/>13	2.6/>38	5.0/>20
1	2	3	4	CND1243	>100	4.1/>24	6.2/>16	1.0/>100	7.2/>14
1	2	3		CND1081	>100	1.5/>66	2.1/>48	3.1/>32	12/8.3
1	2			CND1043	43	4.1/10	3.1/14	5.7/7.5	6.9/6.2
1		3		CND1244	21	1.7/12	2.9/7.2	1.6/13	4.4/4.8
1				CND0975	44	1.0/44	5.5/8	>50/<1.0	>50/<1.0
	2			CND1057	23	3.3/7.0	1.0/23	2.9/7.9	10/2.3
			4	CND0968	29	>25/<1.2	6.4/4.6	6.2/4.7	1.1/26
				CND1020	25	3.9/6.4	13/1.9	3.4/7.3	19/<1.3
				CND1172	15	7.3/2.1	10.4/1.4	4.8/3.1	7.5/2.0

SI is determined by the formula: CC_50_/EC_50_.

## Discussion

From the time of its re-emergence 60 years ago, dengue has spread across the globe, placing nearly 40% of the world's population at risk of infection. Coincidentally, the geographical distribution of the four serotypes has also expanded, with all serotypes reported to co-circulate in most of the dengue-endemic countries [7]. This has serious implications in the rise of DHF/DSS cases, as it is presently understood that antibody-dependent enhancement combined with elevated cytokine responses resulting from subsequent infection with a heterologous serotype are involved in disease severity [35]. The most advanced dengue vaccine candidates try to address this issue by constructing chimeric YF/DENV virus (ChimeriVax-DEN), incorporating the prM and E genes of each DENV serotype in the yellow fever (YF) 17D backbone, and used these to prepare tetravalent cocktails [36]. However, while the tetravalent ChimeriVax-DEN vaccine has shown good immunological responses in clinical trials [17], its long-term safety and efficacy has not been fully established. In contrast to the dengue vaccine approach, therapeutic drug approach circumvents the immunopathological complication of dengue, and directly addresses the acute viral infection. The work reported here describes the development of a high-throughput/high-content assay screening for potential anti-dengue drugs using image-based quantitation of inhibition of dengue virus infection *in vitro* as a measure of antiviral activity. This dengue HT/HCA was used to screen 4,000 small molecules from the BioFocus kinase inhibitor library against DENV1-4, and revealed a number of compounds that inhibit more than 80% infection of all 4 serotypes of dengue *in vitro*. More than 60% of these compounds were also found to inhibit more than 80% infection of HCV genotype 2a infection. Interestingly, most of the compounds belonging to the 4-(1-aminoethyl)-*N*-methylthiazol-2-amine cluster exhibited measurable antiviral activities against dengue viruses in Huh-7.5, but did not demonstrate strong inhibition of HCV genotype 2a infection in the same host cells.

Recently, high throughput assays (HTA) have been used to find several drug candidates with anti-dengue properties [37]. Structure-based dengue virtual screening (i.e. *in silico* high-throughput screening or HTS) is a target-based HTA that analyzes the binding potential of chemical compounds against a target dengue viral protein. Using combinatorial libraries and docking programs that predict the chemical interactions, binding potentials of the compounds with known crystal structures and associated ligands of the target protein are computed. This approach has been used extensively in discovering potential inhibitors of DENV E protein binding and fusion [38], [39] NS5 *2′O-Methyltransferase* [40], NS3 protease [41], [42] and its complex, NS2B/NS3protease (NS2B/NS3pro) [43]. Another target-based HTS approach using enzymatic assay has identified BP2109 as a potential inhibitor of NS2B/NS3pro complex [44]. One drawback of *in silico*-based HTS is that predicted chemical interactions do not take into account other biological factors. This often results in selection of compounds with high binding properties *in silico*, but weak activities once tested *in vitro*. Similarly, target-based enzymatic assays are performed in a highly controlled environment that facilitates optimum enzymatic function of the target, which can be dramatically different from its biological setting. Thus, compounds found to be highly active against the target using this approach may not exhibit the same effect when tested using a cell-based assay, since the assays do not factor in cellular uptake, availability of the target, and other environmental conditions [45]. The *in silico* and target-based HTA approaches are designed to find active compounds against a specific target. While this helps to simplify the process of identifying the probable mechanism of action, it is inherent in the assays to exclude compounds that may be active against other targets involved in viral infection. Hence, their application to the comprehensive screening of active compounds against viral infection is limited.

In contrast to target-based HTA, cell-based HTA is more robust as it covers a wider aspect of the viral infection process. This type of HTA uses either infectious viruses to follow one or multiple rounds of viral infection, or viral replicons to observe the events surrounding viral replication. Cell-based dengue HTA takes advantage of the various intracellular and intercellular events that occur during viral infection. Hence, antiviral properties can be attributed to either compound activity against viral or cellular targets. Cell-based flavivirus immunodetection (CFI), which measures viral protein expression after infection by ELISA, and luciferase reporter viral replicon assay were used to identify inhibitors of viral RNA synthesis namely, the adenosine nucleoside inhibitor NITD008 [14], NS4B inhibitor NITD-618 [46] and NITD-982, an inhibitor of host dihydroorotate dehydrogenase (DHODH) [47]. A modified type of the viral replicon assay using dengue-1 virus-like particles (DENV1-VLP) assembled by packaging the dengue viral replicon tagged with a *Renilla luciferase 2A* reporter gene (*Rluc*2A) in DENV1 structural proteins generated using the Semliki Forest Virus (SFV) expression system was reported to be useful in identifying inhibitors of dengue viral entry, translation and replication [48]. In addition to the expression of viral proteins during infection or replication, other indications of viral infection can be used to assess compound activity such as cell death and reduced metabolic activity. A dengue cytopathic effect (CPE)-based HTA that uses luminescence assay to determine cellular viability by measuring cellular ATP was previously reported [49]. These cell-based HTA are described as “single-readout” assays, since a single value is generated during the assay corresponding to the effect of a particular treatment.

Image-based high-content assay (HCA) is also a form of cell-based assay. Unlike CFI and replicon-based luciferase reporter assays however, it requires more sophisticated equipment like a high-throughput confocal microscope for acquiring images and special software for analyzing image data. When adapting image-based HCA for high-throughput screening, it is more labor intensive and requires more stringent criteria for data analysis. Nonetheless, image-based HCA has one clear advantage over single-readout assays – the amount of information that can be generated from images of a single treatment is not limited to a single value. Aside from the degree of viral infection and cell viability, other interesting information can be extracted from images such as morphological changes in host cell, protein localization, among others [50]. Like other cell-based HTA, image-based assay can be used to screen compounds with diverse modes of activity. This was demonstrated in an image-based HCA screening of 5,362 compounds with diverse chemical structures against DENV2, revealing 73 active compounds, most of which have previously characterized cellular interactions [30]. Dasatinib, a c-Src kinase inhibitor that disrupts the assembly of dengue virions in virus-induced membranous replication complexes, was also identified after screening a kinase inhibitor library using image-based HCA [51].

The image-based dengue HT/HCA developed in this study was used to screen 4,000 compounds belonging to the BioFocus kinase inhibitor library. Primary screening against all four dengue serotypes required 16,000 experiment points (4,000 compounds×4 dengue serotypes), excluding the positive and negative controls. The entire primary screening took 11 days in total: 3 days for expansion of Huh-7.5 from a single T175 tissue culture (TC) flask to 8 T175 TC flasks, 4 days for host cell plating and virus infection in the 384-well plates containing the compounds, 2 days for image acquisition, 1 day for image analysis using the IM platform, and 1 day for data analysis. Two previous image-based screening campaigns for dengue antivirals were conducted with lab-adapted DENV2 (New Guinea C) whole virus [30], [51]. One of these campaigns [51] further investigated the hit compounds by testing the antiviral activities against other lab-adapted dengue serotypes. Our image-based dengue HT/HCA screening campaign differs from the previous image-based HTA in three aspects: First, we used a novel target-focused chemical library (BioFocus kinase inhibitor library) whose collection of small molecules has not been thoroughly screened and characterized. Second, the compounds were screened against low passage strains of field isolated dengue viruses (with the exception of DENV4 tvp360), which allows the identification of compounds that may be active towards prevalent strains. Third, the entire 4,000 compound subset of the BioFocus kinase inhibitor library was screened against all four dengue serotypes. By screening all the compounds of the library against the four dengue serotypes, we can identify novel compounds that may be active against all four serotypes or specific toward any of the serotypes. Such findings can have biological implications on the differences between the viral infection process of the 4 serotypes at the cellular and molecular level. This offers an advantage over the primary screening using the DENV2 serotype and subsequent confirmation of activity with the other serotypes since the latter is already biased towards compounds active against DENV2, resulting in the “loss” of potential hit compounds that do not inhibit this particular serotype.

Screening of the BioFocus kinase inhibitor library using our image-based dengue HT/HCA resulted in the identification of 4 major clusters exhibiting inhibitory properties against dengue virus infection *in vitro*. Among them, one cluster consisting of 11 compounds having a 2-aminothiazole as a core scaffold, showed antiviral activities of varying degrees against the infection of DENV1, DENV2, DENV3, DENV4 in the human hepatoma cell line Huh-7.5. The inability of these compounds to inhibit dengue infection in C6/36 initially suggests that the target is most likely a factor involving dengue virus infection in human cells. However, this discrepancy may also be attributed to other factors, such as difference in membrane permeability between the two different host cells or molecule uptake of the compounds into the host cell. Such differences in the physiology between the human and insect cells may affect the efficacy of these compounds in inhibiting dengue viral infection, but has not been thoroughly investigated in this study. Interestingly, none of the hit compounds from the 2-aminothiazole cluster significantly inhibited the infection of HCV and CHIKV in hepatoma cells, suggesting that the inhibitory property is more specifically directed towards dengue virus infection.

One major drawback when using cell-based assays in high-throughput screening is the effect of toxicity to the host cells, and by extension, viral infection. Compound toxicity can have a profound effect in the viral infection process, and may lead to inaccurate assessment of the antiviral activity. Since the HTS is conducted using only a single concentration of the compounds, it is impossible to avoid encountering those that exhibit moderate to high level of toxicity. Hence, a confirmatory assay that tests a range of concentration is necessary to verify if these hit compounds indeed have antiviral activities. In addition, secondary assays are used to confirm compound activity and predict the mechanism of action. For cell-based assays that utilize image-based technology, determining compound toxicity with high certainty is more difficult. In the absence of biological markers that detect mitochondrial activity, cell apoptosis, cell starvation, the only indicator of compound toxicity is the relative cell number compared with non-treated controls. This can be misleading if the toxicity does not result in abolition of the cells or degradation of the cell nuclei since the cell number will not reflect the actual number of viable cells. Thus, it is essential to confirm compound toxicity by measuring production of ATP or relative oxygen species as an indicator of cell viability [52].

**Figure 5:**
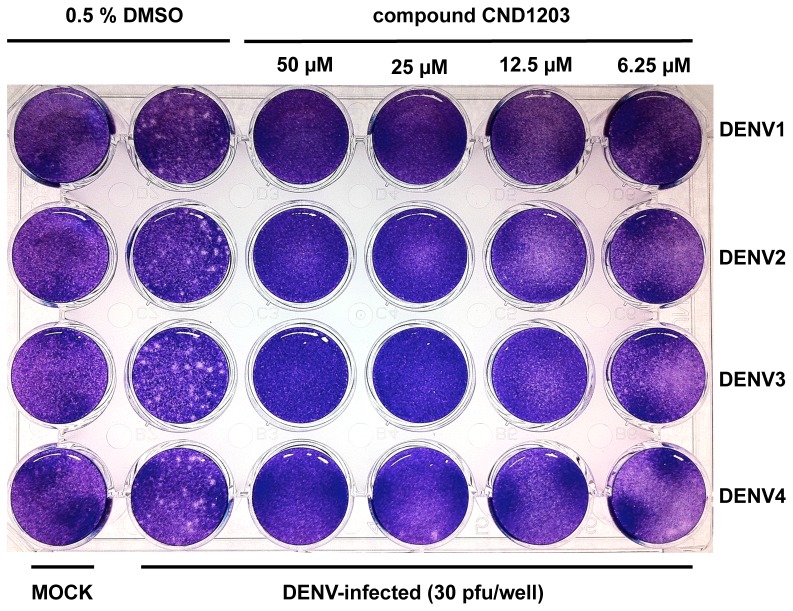
Plaque reduction assay. Huh-7.5 cells grown in 24-well tissue culture plate were inoculated with 30 pfu DENV1, DENV2, DENV3 and DENV4 per well and incubated for 7 days in the presence of compound CND1203 at various concentrations. MOCK-infected wells were inoculated with VM only.

Time-of-addition assay, a strategy to determine the stage of inhibition during the viral infection cycle, has been used to characterize the mode of action of some inhibitors of dengue viral entry (NITD Compound 6), viral replication (NITD-982) and early translation (NITD-2636) [39], [47], [53]. The inhibitor is added at different time points during viral infection and monitored for expression of the viral proteins, replication of the genome or production of infectious progeny virions. Among the 2-aminothiazole hit compounds identified in this study, CND1203 exhibited a strong antiviral activity against all 4 dengue serotypes, and blocked the formation of dengue virus plaques in Huh-7.5 at 25 µM in the plaque reduction assay (Figure 5). Compound CND1203 was used for the time-of-addition assay, adding 25 µM at different time points (−2 hpi, 0 hpi, 0.5 hpi, 1 hpi, 2 hpi, 4 hpi) of the dengue virus infection (M.O.I. 5) in Huh-7.5. In contrast to the strong inhibition of compound CND1203 against dengue viruses in the plaque reduction assay, none of the treatments inhibited dengue infection in the time-of-addition assay, suggesting that the compound does not interfere with viral entry (data not shown). Aminothiazole-based compounds have previously been implicated in the inhibition of HCV replication by binding to an allosteric site on the viral polymerase [54]. However, the structure of these active anti-HCV compounds differ from the 2-aminothiazole hit compounds reported in this study in terms of the substitutions on the scaffold. As a consequence, it is unlikely that our hit compounds interact with the viral polymerase.

Although compound CND1203 did not inhibit DENV entry, its ability to inhibit the spread of DENV infection and formation of virus plaques in Huh-7.5 clearly suggest that the mode of action is at the post-entry stages. Two kinase inhibitors were previously reported to block virus assembly of dengue virions: Dasatinib, a thiazolyaminopyrimidine that inhibit c-Src protein kinase, did not interfere with dengue RNA replication, but disrupted the proper assembly of dengue virions within virus-induced cell membranous replication complex [51]. SFV785, a trifluorinated *N*-methylanaline derivative that selectively inhibits NTRK1 and MAPKAPK5 kinase activity, altered the distribution of structural envelope protein from the reticulate network to enlarged discrete vesicles, consequently affecting the co-localization with the DENV replication complex and disrupting the assembly of progeny virions [55]. Interestingly, the 2-aminothiazole hit compounds identified in this study shares the aminothiazole moiety of Dasatinib, as well as the rings on each of the molecule. Based on the structural similarity with Dasatinib, c-Src kinase may be a candidate target of the 2-aminothiazole hit compounds, which would imply a post-genomic replication mode of action. Further investigation is necessary to support this hypothesis.

The persistence of dengue outbreaks around the world, and the lack of an available dengue vaccine reinforce the need to find and develop therapeutic drugs to address this major health concern. The use of HT/HCA screening technologies can expedite the drug discovery of potential dengue antivirals by facilitating the screening of large chemical libraries. The work reported here features an innovative image-based HT/HCA system that can be used as a reliable tool in screening for antiviral compounds against all four DENV serotypes. Furthermore, the compounds identified in the present study can serve as a potential starting point for the development of dengue antivirals.

## Supporting Information

Figure S1 Development of the dengue HT/HCA. Workflow diagram of the dengue HT/HCA from assay development to the actual screening and hit confirmation (A). Flow chart of the immunofluorescence assay that includes image acquisition and analysis (B).
(TIF)

Figure S2 Infection Kinetics of DENV in Huh-7.5. Percentage of dengue-infected cells resulting from Den1 BR/90, BR DEN2 01-01, BR DEN3 290-2 and DEN4 TVP360 infection at various M.O.I. and incubation period.
(TIF)

Figure S3 Assay validation of the image-based dengue HT/HCA. Scatter plot and calculated Z'-factors of the dengue HT/HCA for DENV1, DENV2, DENV3 and DENV4 infection in Huh-7.5. Dots represent DENV-infected (red) and MOCK-infected (blue) Huh-7.5 based on image analysis using in-house IM platform. Area under the black and green dotted lines represents the variability of the DENV infection and MOCK infection controls, respectively. The arrows represent the degree of separation (Z'-factor) between the two controls.
(TIF)

Figure S4 Profiling of primary hits from the dengue HT/HCA of the BioFocus kinase inhibitor library. *Left*: Dendogram showing structural similarity of dengue primary hits based on tanimoto similarity index (http://chemmine.ucr.edu). *Right*: Percent inhibition of the 157 primary hits at 10 µM against DENV1, DENV2, DENV3 and DENV4 infection in Huh-7.5 and C6/36, HCV genotype 2a infection in Huh-7.5 and CHIKV infection in HuH-7. Shades indicate range of activity: <50% (red), 50%–90% (yellow), >90% (green). The selected hit compounds belonging to the 4-(1-aminoethyl)-N-methylthiazol-2-amine cluster are marked (★).
(TIF)
