# Supplementary figures and images for: High Content Screening of a Kinase-Focused Library Reveals Compounds Broadly-Active against Dengue Viruses
Source: PLoS Negl Trop Dis. 2013 Feb 21;7(2):e2073. doi: 10.1371/journal.pntd.0002073 (PMC3578765; doi:10.1371/journal.pntd.0002073)

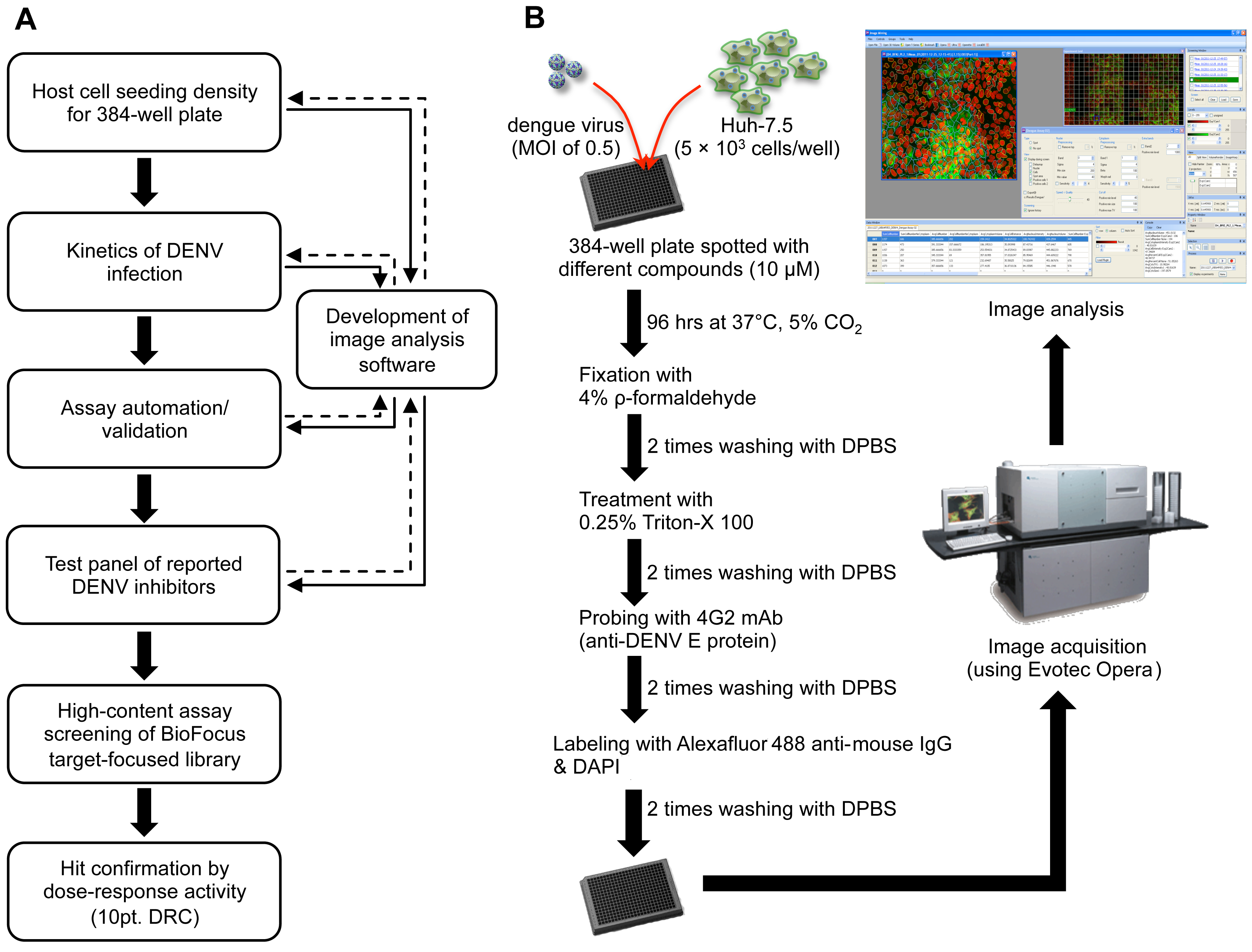

Supplement: Figure S1 — Development of the dengue HT/HCA. Workflow diagram of the dengue HT/HCA from assay development to the actual screening and hit confirmation (A). Flow chart of the immunofluorescence assay that includes image acquisition and analysis (B). (TIF) [file pntd.0002073.s001.tif]

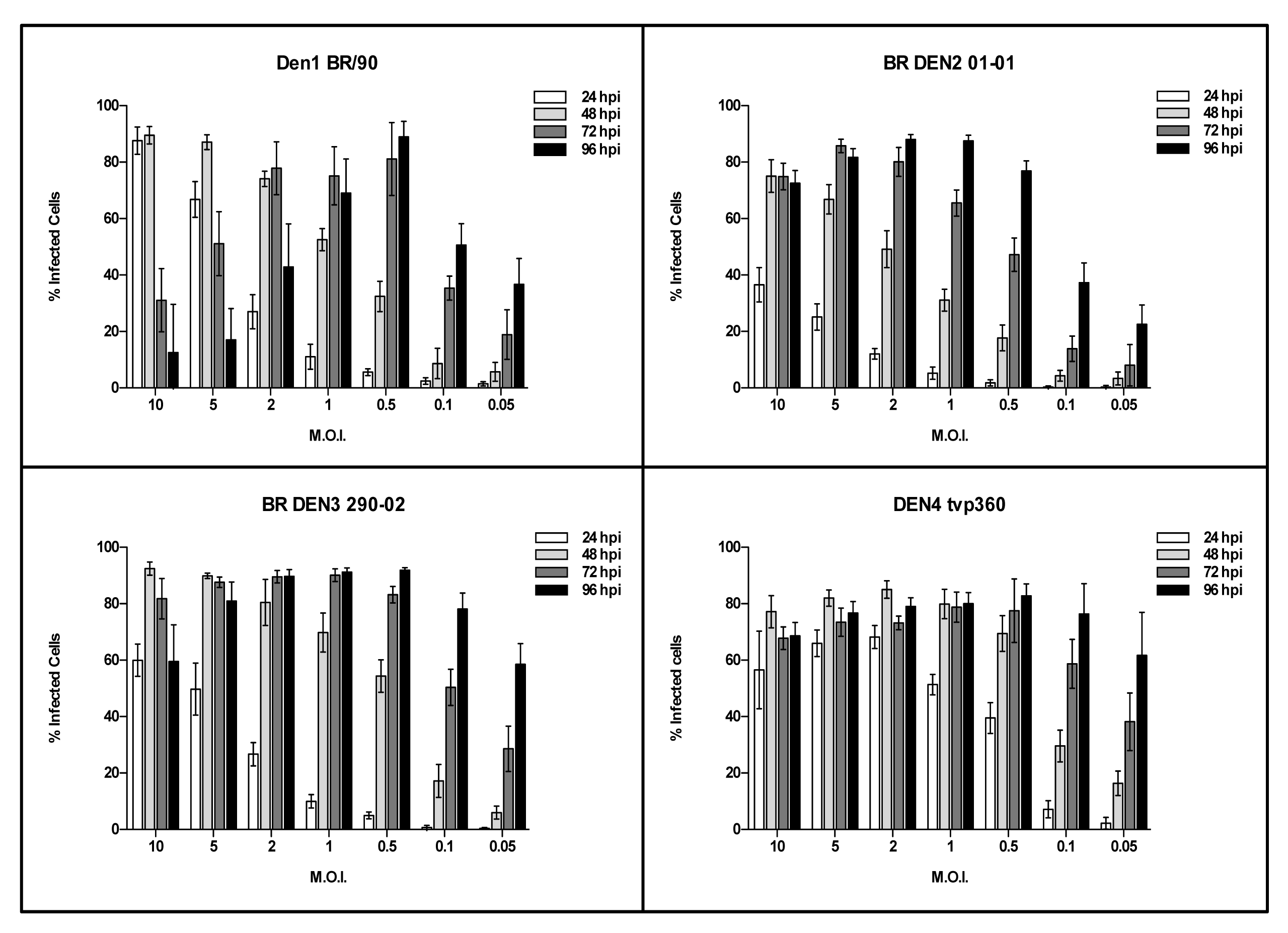

Supplement: Figure S2 — Infection Kinetics of DENV in Huh-7.5. Percentage of dengue-infected cells resulting from Den1 BR/90, BR DEN2 01-01, BR DEN3 290-2 and DEN4 TVP360 infection at various M.O.I. and incubation period. (TIF) [file pntd.0002073.s002.tif]

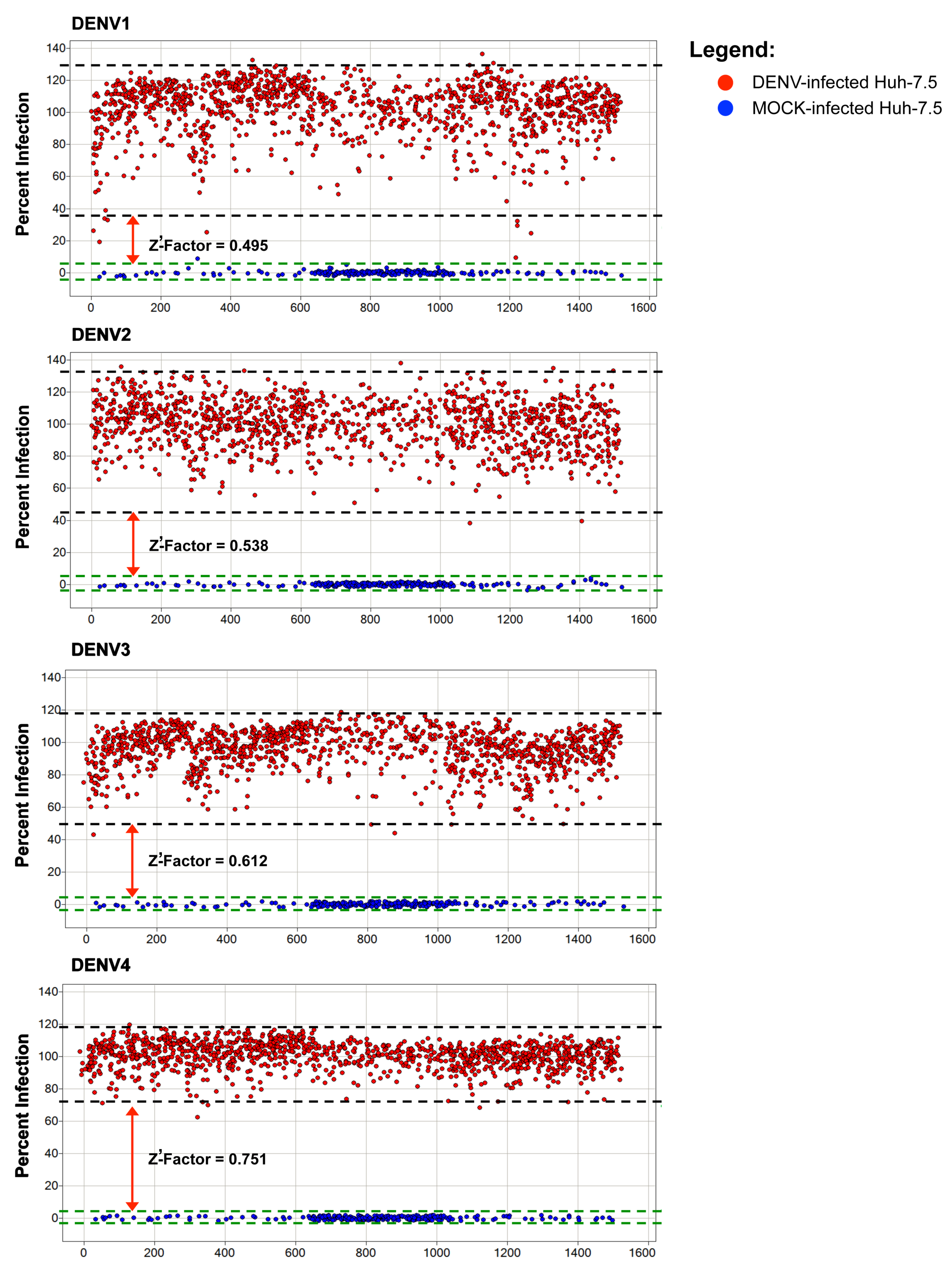

Supplement: Figure S3 — Assay validation of the image-based dengue HT/HCA. Scatter plot and calculated Z'-factors of the dengue HT/HCA for DENV1, DENV2, DENV3 and DENV4 infection in Huh-7.5. Dots represent DENV-infected (red) and MOCK-infected (blue) Huh-7.5 based on image analysis using in-house IM platform. Area under the black and green dotted lines represents the variability of the DENV infection and MOCK infection controls, respectively. The arrows represent the degree of separation (Z'-factor) between the two controls. (TIF) [file pntd.0002073.s003.tif]

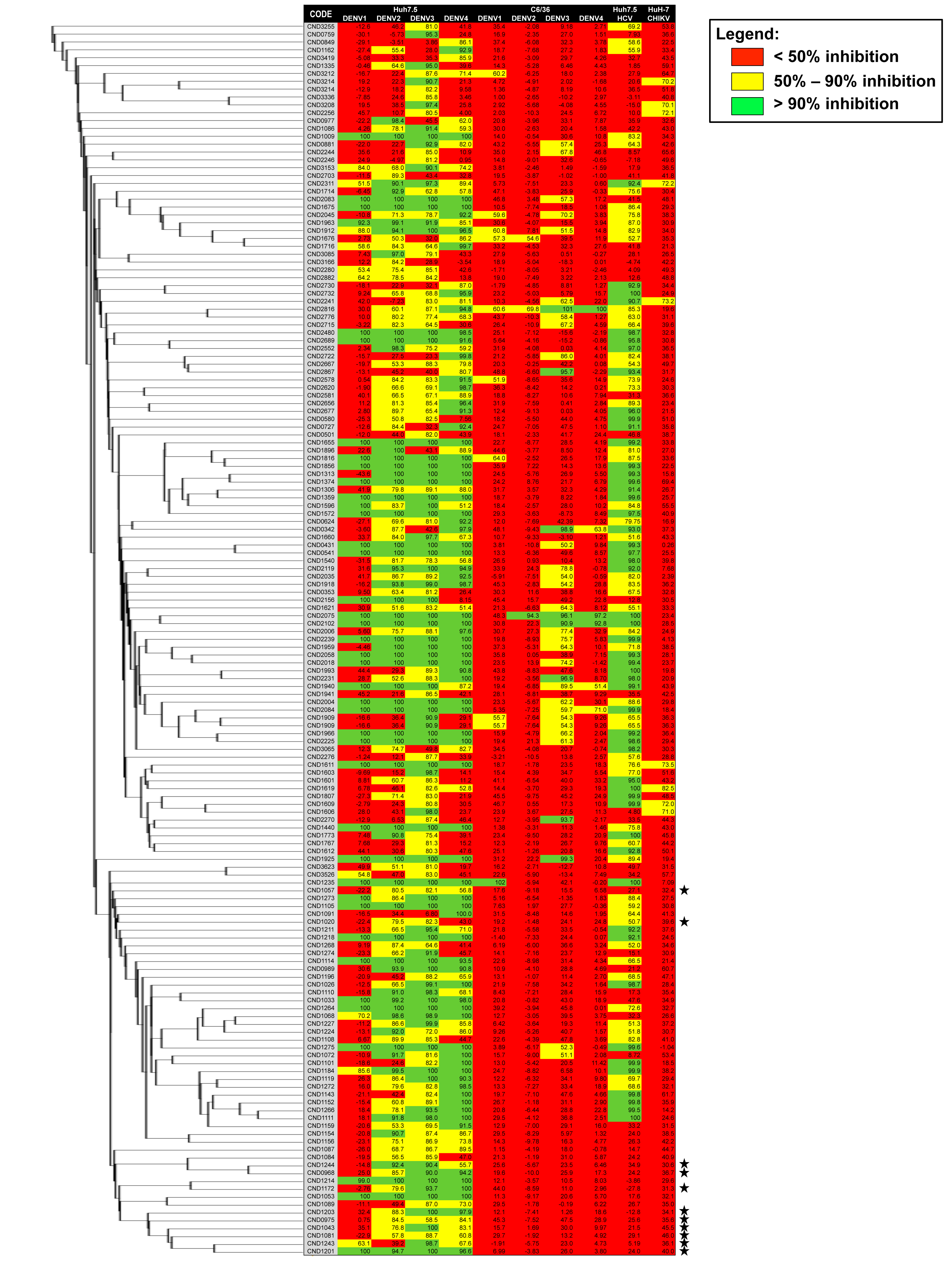

Supplement: Figure S4 — Profiling of primary hits from the dengue HT/HCA of the BioFocus kinase inhibitor library. Left: Dendogram showing structural similarity of dengue primary hits based on tanimoto similarity index (http://chemmine.ucr.edu). Right: Percent inhibition of the 157 primary hits at 10 µM against DENV1, DENV2, DENV3 and DENV4 infection in Huh-7.5 and C6/36, HCV genotype 2a infection in Huh-7.5 and CHIKV infection in HuH-7. Shades indicate range of activity: <50% (red), 50%–90% (yellow), >90% (green). The selected hit compounds belonging to the 4-(1-aminoethyl)-N-methylthiazol-2-amine cluster are marked (★). (TIF) [file pntd.0002073.s004.tif]
